# The bioactivity of soluble Fas ligand is modulated by key amino acids of its stalk region

**DOI:** 10.1371/journal.pone.0253260

**Published:** 2021-06-17

**Authors:** Osamu Kajikawa, Raquel Herrero, Yu-Hua Chow, Chi F. Hung, Gustavo Matute-Bello

**Affiliations:** 1 Center for Lung Biology, Division of Pulmonary and Critical Care Medicine, Department of Medicine, University of Washington, Seattle, Washington, United States of America; 2 Medical Research Service, VA Puget Sound Healthcare System, Seattle, Washington, United States of America; 3 Instituto de Salud Carlos III, Hospital Universitario de Getafe and CIBER de Enfermedades Respiratorias, Madrid, Spain; Universite Paris-Sud, FRANCE

## Abstract

We have previously reported that the 26-amino acid N-terminus stalk region of soluble Fas ligand (sFasL), which is separate from its binding site, is required for its biological function. Here we investigate the mechanisms that link the structure of the sFasL stalk region with its function. Using site-directed mutagenesis we cloned a mutant form of sFasL in which all the charged amino acids of the stalk region were changed to neutral alanines (mut-sFasL). We used the Fas-sensitive Jurkat T-cell line and mouse and human alveolar epithelial cells to test the bioactivity of sFasL complexes, using caspase-3 activity and Annexin-V externalization as readouts. Finally, we tested the effects of mut-sFasL on lipopolysaccharide-induced lung injury in mice. We found that mutation of all the 8 charged amino acids of the stalk region into the non-charged amino acid alanine (mut-sFasL) resulted in reduced apoptotic activity compared to wild type sFasL (WT-sFasL). The mut-sFasL attenuated WT-sFasL function on the Fas-sensitive human T-cell line Jurkat and on primary human small airway epithelial cells. The inhibitory mechanism was associated with the formation of complexes of mut-sFasL with the WT protein. Intratracheal administration of the mut-sFasL to mice 24 hours after intratracheal *Escherichia coli* lipopolysaccharide resulted in attenuation of the inflammatory response 24 hours later. Therefore, the stalk region of sFasL has a critical role on bioactivity, and changes in the structure of the stalk region can result in mutant variants that interfere with the wild type protein function *in vitro* and *in vivo*.

## Introduction

The acute respiratory distress syndrome (ARDS) is defined by the sudden onset of bilateral lung infiltrates and impaired gas exchange, in the absence of evidence of left ventricular dysfunction [1, 2]. ARDS is an important clinical problem in the United States, affecting 200,000 patients per year and resulting in death of approximately 75,000 persons [3]. Over the past 20 years the mortality due to ARDS has decreased from approximately 60% to 30–40%, in part because of the discovery that mechanical ventilation with lower tidal volumes is protective; however, mortality remains unacceptably high [4]. Furthermore, there is increasing evidence that survivors suffer significant long-term consequences [5]. Despite these negative outcomes, specific treatments are lacking.

Data from our laboratory and others suggest that the Fas/FasL system plays a role in the pathophysiology of human ARDS and of its animal correlate, acute lung injury (ALI) [6–11]. The Fas/FasL system is comprised of the membrane surface receptor Fas (CD95) and its cognate ligand, FasL (CD178). In the lungs, Fas is expressed in the airway and alveolar epithelia, in fibroblasts and in alveolar macrophages [12]. Binding of FasL to Fas activates signaling pathways that lead to apoptosis and also to cytokine release [13] (S1 Fig). FasL is expressed on cell membranes as a transmembrane protein that can be cleaved to a soluble form–sFasL–that binds its receptor. FasL, soluble or membrane-bound, must form homotrimers in order to activate Fas [14]. We have found that in the lungs, the main cellular targets of sFasL are alveolar epithelial cells, which respond to Fas ligation with both apoptosis and release of pro-inflammatory cytokines [12, 13, 15, 16]. We have also found that sFasL is present in the bronchoalveolar lavage (BAL) fluid of patients with ARDS, that administration of sFasL to mice and rabbits results in lung injury, and that mice lacking Fas have attenuated alveolar inflammation in response to intratracheal lipopolysaccharide (LPS), mechanical ventilation, or viral infection [6, 9–11, 17]. For example, mechanically ventilated mice exposed to LPS develop BAL neutrophilia, which is markedly attenuated in mice deficient in functional Fas (B6.MRL-*Fas*^*lpr*^/J, “*lpr*” mice) [18]. Furthermore, silencing of Fas attenuates lung injury in mice [8]. Thus, the Fas/FasL system is active in the lungs and plays a role in the development of several different models of acute lung injury. In addition to the lungs, in humans mutations in either Fas or FasL are associated with a syndrome of immune dysregulation characterized by cytopenias, predisposition to infections and lymphoproliferative disorders known as “autoimmune lymphoproliferative disorder”, or ALPS [19].

We have previously reported that the biological activity of sFasL in the lungs is dependent on its structure [20]. Specifically, in the lungs sFasL exists in at least two forms: a 152-amino acid short form, consisting primarily of the Fas binding domain and a short “stalk” region, and a 178 amino-acid long form, consisting of the Fas binding domain plus a longer stalk region [20]. The long form can be cleaved into the short form by proteases such as MMP7 [21]. Interestingly, despite the fact that the short form contains the binding domain, only the long form of sFasL is capable of inducing lung injury in mice, which is characterized by increased alveolar permeability, neutrophilic alveolitis, and apoptosis of alveolar epithelial cells [20]. Importantly, the long form of sFasL is the major form present in BAL fluid from patients with ARDS [20].

Because both the long and short forms of sFasL contain the Fas binding domain, we hypothesized that the reason for the difference in bioactivity is a structural change resulting from the presence or absence of the stalk region. Therefore, in this study we mutated specific charged amino acid residues in the stalk region and determined whether such mutations affect sFasL activity. We found that the substitution of all 8 charged amino acids of the stalk region resulted in significant loss of function, confirming the critical role of the stalk region in the bioactivity of sFasL. The responses seen in LPS-treated mice exposed to the mutated form of sFasL (mut-sFasL) were similar to the published phenotype of ventilated mice lacking functional Fas (*lpr*) following LPS exposure [18].

## Materials and methods

### Antibodies and reagents

To prepare immunoaffinity columns we used mouse anti-human FasL monoclonal antibodies, clone 100419 (R&D System Cat# MAB126, RRID:AB_2246667, Minneapolis, MN). For the Fas-FasL binding assay, we used recombinant human soluble Fas receptors (PeproTech, Rocky Hill, NJ) and biotinylated goat anti-human FasL polyclonal antibodies (PeproTech Cat# 500-P184bt-50ug, RRID:AB_148184). Mouse anti-human Fas (CD95) monoclonal IgM antibody, clone CH-11 and its isotype control antibody, clone 3D12, were obtained from MBL, Nagoya, Japan (MBL International Cat# SY-001, RRID:AB_591016 and Cat# M077-3, RRID:AB_593057, respectively). FLAG tagged mut-sFasL was detected using HRP anti-FLAG monoclonal antibody (Sigma-Aldrich, Cat# A85920). Unless otherwise indicated, all other chemicals were purchased from Sigma-Aldrich (St. Louis, MO).

### Cells and cell culture

To test Fas responses *in vitro* we used the highly-Fas sensitive cell line, Jurkat. Jurkat cells are human T lymphocyte cells first isolated from the peripheral blood of a 14 year old boy with acute T cell leukemia [22]. Jurkat cells are exquisitely sensitive to Fas ligation and are commonly used as a model cell in studies involving the Fas/FasL system; here they are used as such, rather than as a model of T-cell responses. Our Jurkat cells were purchased from ATCC (Clone TIB-152) and grown at 37°C and 5% CO_2_ in RPMI-1640 medium supplemented with 10% heat-inactivated fetal bovine serum. Cells were authenticated using short tandem repeat analysis. Mycoplasma status was weakly positive by PCR (Venor GeM Mycoplasma Detection Kit, Sigma-Aldrich, Cat# MP0025). The Jurkat data was confirmed in primary human lung epithelial cells and the murine alveolar epithelial cell line LA4, which are described below.

Small airway epithelial cells (SAEC) are primary human cells isolated from the 1-mm bronchiole area of normal human lung tissue, and were purchased from Lonza (Walkersville, MD, catalogue number CC-2547, lot number 0000669507). All cells used came from a single donor and confirmed mycoplasma-free by the vendor. Cells were grown at 37°C, 5% CO_2_ in small airway epithelial basal medium (SAGM, Lonza, Cat# CC-3119 CC-3119) supplemented with SAGM SingleQuots (Lonza, catalogue number CC-4124) and used between passages 2 and 4.

Mouse lung epithelial LA-4 cells were obtained from ATCC (catalogue number CCL-196, lot number 3404534). This cell line is derived from a A/He mouse lung adenoma [23]. Our lot was certified as mycoplasma-free by the vendor. The cells were grown at 37°C, 5% CO_2_ in F-12K medium supplemented with 15% heat-inactivated fetal bovine serum.

FreeStyle 293-F cells are human embryonic kidney cells used as a mammalian expression system, and were obtained from Invitrogen (catalogue number P/N 51–0029, lot number 1038913). The cells were grown in FreeStyle 293 expression medium at 37°C and 8% CO_2_ while shaking at 135 rpm. Mycoplasma status was negative as determined by PCR.

### Cloning of human sFasL cDNA and site-directed mutagenesis

**Fig 1:**
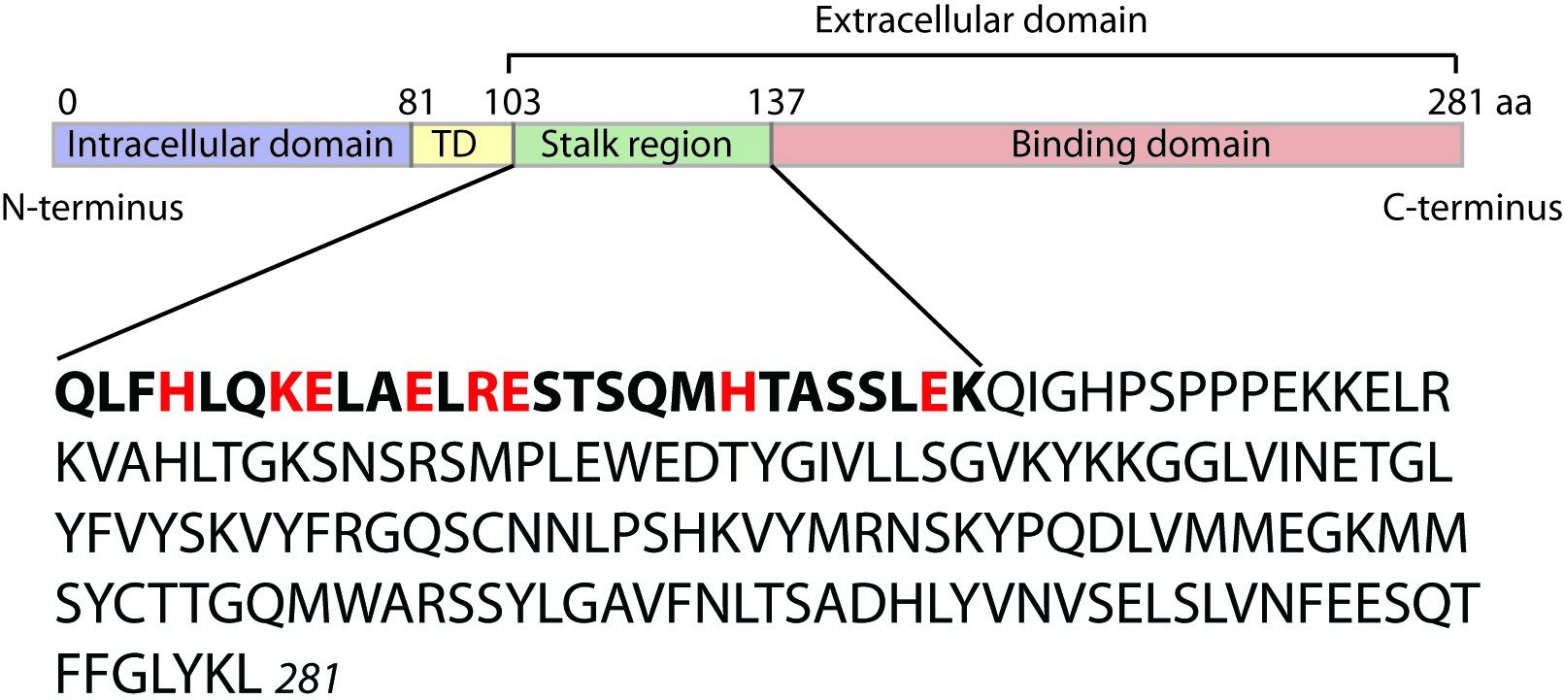
Fas ligand structure and modifications. Fas ligand is a 281 amino acid protein that contains an intracellular domain, a transmembrane domain (TD), a “stalk region”, and the binding domain. Fas ligand can be cleaved my metalloproteinases at amino acid 103 to form a soluble form that retains activity. Soluble Fas Ligand (sFasL) can be further cleaved at amino acid 128 into a “short” soluble form that is inactive. We mutated all the charged amino acids of the stalk region to non-charged alanines (shown in red).

Human sFasL cDNA sequences coding amino acids 103 to 281 were amplified with DNA polymerase (Easy-A High-Fidelity PCR Cloning Enzyme, Agilent Technologies, Cat# 600400, La Jolla, CA) using cloned human sFasL cDNA (ATCC 10659567) as a template and cloned into pSecTag/FRT/V5-His-TOPO mammalian expression vector (Invitrogen, Cat# K6025-01, Carlsbad, CA) (Fig 1). Although this vector had a V5-His tag coding region on the 3’-end of the cloning site, the expressed sFasL was the native form because the amplified sFasL cDNA contained appropriate stop codons. Site-directed mutagenesis in the plasmid encoding sFasL cDNA was performed using a QuikChange II site-directed mutagenesis kit (Agilent Technologies, Cat# 200523–5). FLAG-tag coding cDNA was cloned into 3’-end of 281th amino acid coding sequence of mut-sFasL cDNA sequence in frame to produce mut-sFasL-FLAG expression plasmid (pSec mut-sFasL-FLAG). The sequences of the cloned pSecTag/cDNA-sFasL plasmids were confirmed by commercial DNA sequencing services by Eurofins MWG/Operon (Huntsville, AL).

### Expression of sFasL protein in mammalian cells

Suspensions of human embryonic kidney cells (FreeStyle 293-F cells, Invitrogen) were transfected with pSecTag/cDNA-sFasL encoding the wild type human sFasL cDNA and the 8-site mutated human sFasL cDNA using 293fectin lipofection reagent (Invitrogen) and incubated in a humidified incubator at 8% CO2, 37°C with the FreeStyle293 expression serum-free medium (Gibco, Carlsbad, CA, Cat# 12338–018) while shaking. After 6 days of incubation, cell supernatants were harvested, filtered with a 0.22 μm nitrocellulose membrane and stored at –20°C until used. The FasL protein was purified using immunoaffinity columns (HiTrap NHS-activated HP, GE Healthcare, Piscataway, NJ, Cat# 17071601), in which mouse monoclonal antibodies to human FasL (R&D System, Clone: 100419, Minneapolis, MN) were immobilized on agarose beads. The concentrations of purified human sFasL proteins were measured by a human sFasL ELISA kit (MBL, Nagoya, Japan, Cat# 5255) following the manufacturer’s instructions.

### Biotinylation of sFasL

Immunoaffinity purified WT-sFasL was biotinylated with No-Weight Format EZ-Link Sulfo-NHS-LC-Biotin (Pierce, Rockford, IL, Cat# 21327) following the manufacturer’s instructions. To capture biotinylated WT-sFasL in the mixtures of biotinylated WT-sFasL and mut-sFasL-FLAG, Streptavidin-variant (Strep-Tactin XT) coated 96-well plates (iba, Gottingen, Germany, Cat# 2-4101-001) were used.

### Measurements of apoptotic cascade activation

Apoptotic cascade activation was assessed by determining caspase 3/7 activity and Annexin V externalization. Caspase-3/7 activity was measured using a commercial substrate (Caspase-Glo 3/7 Assay, Promega, Madison, WI, Cat# G8090) following manufacturer’s instructions. Annexin V was measured with a luminescent commercial assay that allows for measurement of annexin V externalization in adherent cells (RealTime-Glo Annexin V assay, Promega, Madison, WI, Cat# JA1011). Luminescence was measured with a Synergy H4 Microplate Reader (BioTek, Winooski, VT).

### Animal studies

Animal protocols were approved by the institutional animal care and utilization committees of the University of Washington and the Veterans Affairs Puget Sound Healthcare System (protocols number 4124–04 (UW) and 0902 (VA). Mice were housed in conventional cages with ad libitum access to tap water and standard chow, in humidity-controlled (50%) and temperature-controlled (22°C–23°C) rooms with 12 hours/12 hours light/dark cycles. In the morning, male C57BL/6J mice (Jackson Laboratories, Bar Harbor ME) weighing 24–30 g were anesthetized with 4% inhaled isoflurane and randomized to treatment for one oropharyngeal instillations of either PBS, or LPS, 2.5 μg/g (Sigma-Aldrich, St. Louis MO)(Time = 0 hr). The PBS group was instilled first to avoid cross-contamination with LPS. The mice were allowed to recover from anesthesia and given full access to food and water. Some of the mice received oropharyngeal instillations of mut-sFasL at 4 hr and were euthanized at 24 hr (“early” group). Other mice received oropharyngeal mut-sFasL at 24 hours and were euthanized at 48 hr (“late” group). In both cases, mut-sFasL doses were 0, 30 and 100 ng/mL. The number of mice used per group was 6, and the total number of mice was 54.

Following instillations, the mice were monitored for signs of discomfort every 15 minutes during the first hour, and then every four hours during daytime. We avoided routinely checking on the mice at night as that could be disruptive for all of the mice and cause stress. The primary monitoring parameters were loss of prone posture, loss of movement with mild physical stimulation, weight loss of ≥ 20% (weight was measured once per day) and severe respiratory distress. Secondary monitoring parameters included ruffled fur, nasal or ocular discharge and an abnormal respiratory pattern. The presence of one major parameter or two minor parameters was considered an indication for euthanasia, but no mice met these criteria.

At the end of the experiments the mice were euthanized with intraperitoneal injections of Beuthanasia-D (1.56 mg/g of pentobarbital component) and exsanguinated by severing the inferior vena cava. The thorax was opened rapidly, the trachea was cannulated with a 20-gauge catheter, the left hilum was clamped and the left lung removed and flash-frozen in liquid nitrogen for homogenization, and the right lung was subjected to bronchoalveolar lavage (BAL) as previously described [17]. In some animals, the right lung was also fixed in 4% paraformaldehyde at an inflation pressure of 15 cm H_2_O and embedded in paraffin for subsequent histological studies.

### BAL and lung homogenate measurements

Total cell counts in the BAL fluid were performed with an automated cell counter (Cellometer, Nexcelom, Lawrence, MA). Differential counts were performed on cytospin preparations using the Diff-quick method (Fisher Scientific Company L.L.C., Kalamazoo, MI). Total protein concentrations were measured with the bicinchoninic acid method (BCA assay, Pierce, Rockford, IL). Tissue polymorphonuclear leukocytes (PMNs) were assessed by myeloperoxidase (MPO) activity measured in lung homogenates using the Amplex red peroxidase assay kit (Invitrogen, Carlsbad, CA). BAL fluid cytokines were measured using a fluorescent-bead based luminex multiplex reader (Bioplex 200, Biorad, Hercules, CA) and multiplex cytokine beads (Invitrogen, Carlsbad, CA).

### Statistical analysis

Quantitative variables were expressed as mean ± SD. Analysis of multiple groups was done with one-way ANOVA when only one factor was tested, or 2-way ANOVA when two factors were tested. Significance between groups was determined by Sidak’s post-hoc analysis for one-way ANOVA or Dunnett’s analysis for 2-way ANOVA. A p value less than 0.05 was considered statistically significant. The statistical analyses were performed using GraphPad Prism software, version 9.0.0.

## Results

### Mutations of the charged amino acids in the stalk region of sFasL influence its biological activity in vitro

**Fig 2:**
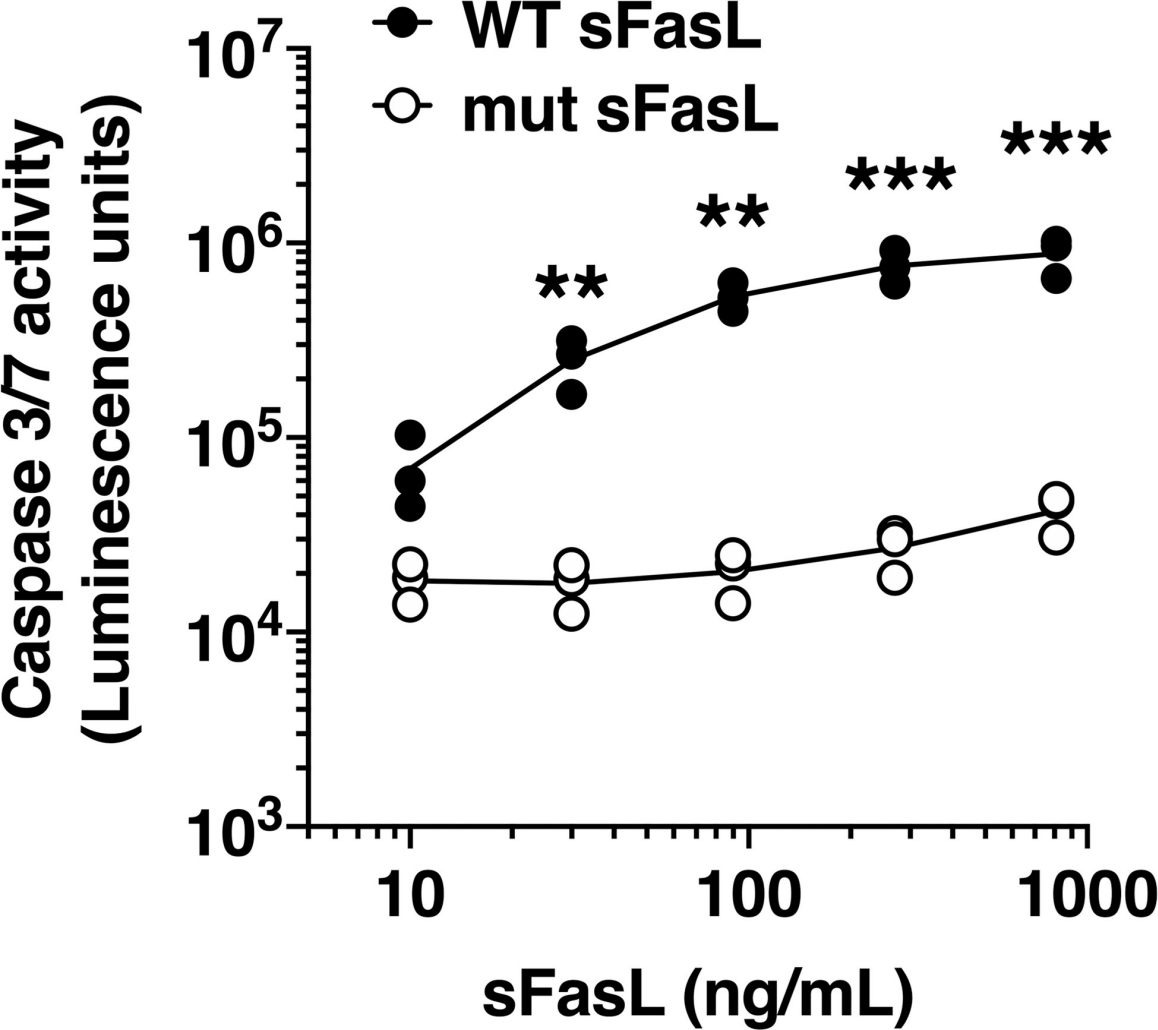
Bioactivity of the sFasL mutant protein. Jurkat cells were incubated for 5 hours with wild type (WT) sFasL or a sFasL mutant (mut sFasL) in which all of the 8 charged amino acids were changed to alanine. Caspase 3/7 activity was measured after incubation with the caspase 3/7 substrate at room temperature for 30 minutes. Data are shown as arbitrary luminescence units. Results show data from three separate experiments, each performed in duplicate. Each dot represents individual data. Data analyzed by 2-way ANOVA with Sidak post-hoc analysis. ** p < 0.01, *** p < 0.001 compared with the respective concentration of mut-sFasL.

To test whether the structure of the stalk region of sFasL influences its biological activity we cloned a mutant sFasL molecule (mut-sFasL) in which all of the 8 charged amino acids in the 26 amino acid stalk region were changed to the non-charged amino acid alanine (Fig 1, S2 Fig). Using the Rosetta Molecular Modeling Suite at the Institute for Protein Design (https://rosbetta.bakerlab.org/), we modeled the structure of the mut sFasL monomer and trimer, illustrating the predicted conformational changes in the stalk region when the charged residues are changed to alanine (S3 Fig). We then tested its ability to activate caspases 3/7, a key step in the apoptosis cascade. We used human Jurkat t-cells because they are highly sensitive to Fas activation and therefore are a good tool to test the Fas/FasL system. We found that the mut-sFasL had significantly reduced caspase 3/7 activity compared to WT-sFasL (Fig 2). The mutant-sFasL retained some bioactivity at the highest concentration tested (810 ng/mL) but its activity was approximately two orders of magnitude less than the wild-type sFasL (p = 0.04 for mutant sFasL at 910 ng/mL vs 10 ng/mL).

### The mut-sFasL attenuates the activity of WT-sFasL

**Fig 3:**
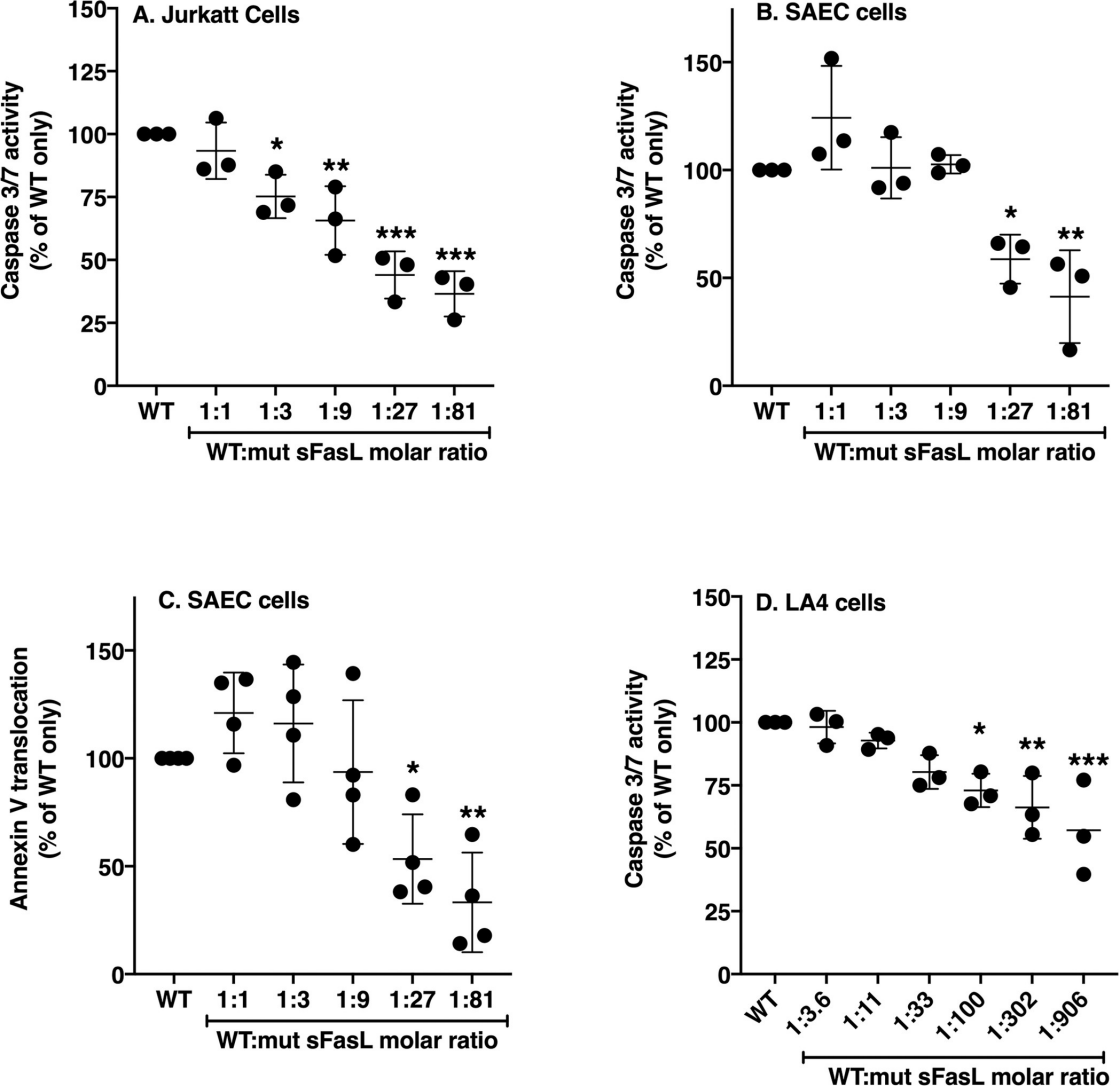
The mut-sFasL has inhibitory properties. Highly Fas-sensitive Jurkat cells (A), primary human lung small airway epithelial cells (SAEC, B) or murine lung epithelial cells (LA4, D) were incubated with serial dilutions of mutated FasL, (mut-sFasL). The 8-site mutated sFasL caused a dose-dependent inhibition of wild type sFasL activity in Jurkat cells (A). The human lung epithelial cells (SAEC) and murine lung epithelial cells (LA4) were less sensitive to WT sFasL, but an inhibitory effect of the mutant protein was detected (B and D). As a confirmatory test, the experiment was repeated using SAEC and an annexin-V translocation assay (C). WT = wild type only (no mutant). Results show data from three or four separate experiments, each performed in duplicate. Each dot represents individual data, lines represent means ± SD. * = P < 0.05; ** = P<0.01, ***P<0.001 when compared to WT only using one way ANOVA with Sidak’s post hoc analysis.

We then investigated caspase 3/7 activation in Jurkat T-cells incubated with different molar ratios of WT and mut-sFasL. We found that the mut-sFasL significantly attenuated WT-sFasL activity at molar ratios greater than 1:3 WT:mut-sFasL (Fig 3A, S4 Fig) then tested the molar mixtures on primary human small airway epithelial cells (SAEC), which are commercially isolated from the distal airways of human lungs. Again, we found that the mut-sFasL inhibited the ability of WT-sFasL to induce caspase 3–7 activity, although only at higher molar ratios (Fig 3B). Also, we repeated the experiment using SAEC and an alternative measurement of apoptotic activation, annexin-V externalization on the cell membrane. We again found that the mut-sFasL attenuated the activity of WT-sFasL at molar ratios of 1:27 or higher (Fig 3C). Finally, we found that mut-sFasL also inhibited the ability of WT-sFasL to induce caspase 3–7 activity in murine lung epithelial cells, although these cells are less sensitive to human FasL (Fig 3D).

### WT-sFasL inhibition likely occurs prior to receptor binding

**Fig 4:**
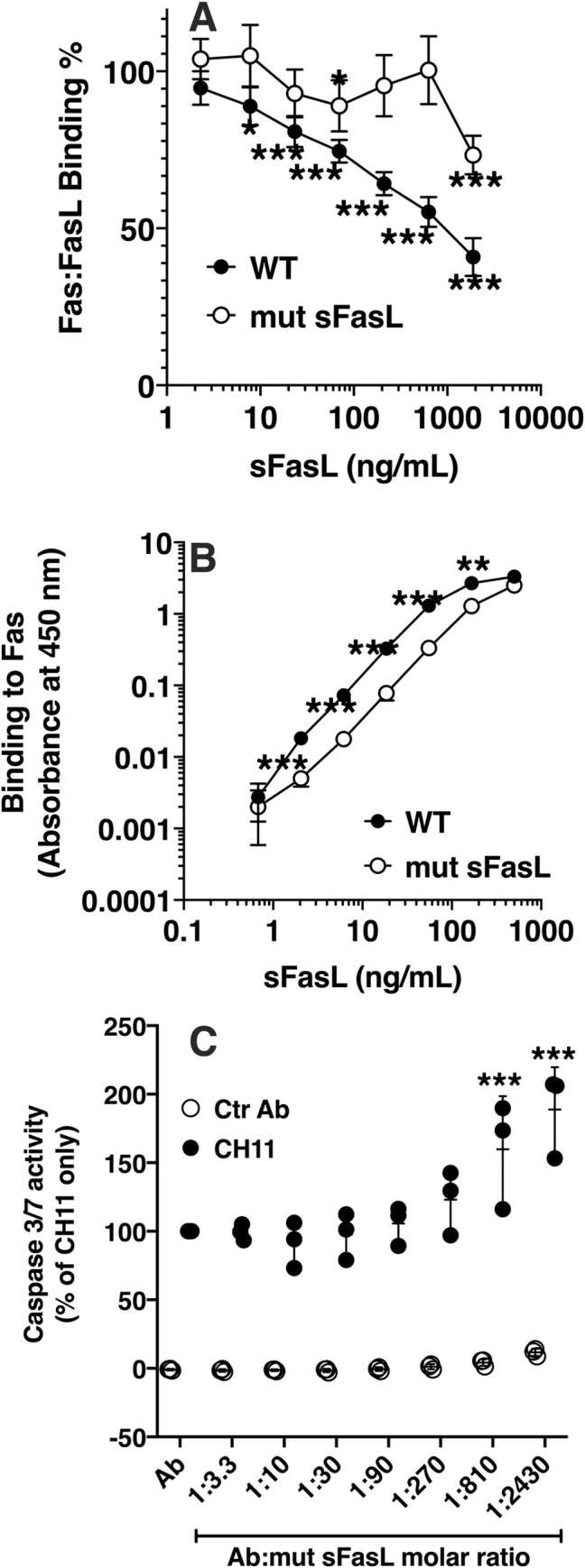
Competition and binding assays. (A) Competition assay. Serial 3-fold dilutions of either WT or 8-site mutated sFasL were mixed with biotinylated WT sFasL (70 ng/ml) and incubated for 2 hours in 96-well plates pre-coated with recombinant human soluble Fas (250 ng/ml). Biotin was detected using HRP conjugated streptavidin; (B) Fas binding assay. Serial 3-fold dilutions of wild type (WT) or mut-sFasL were incubated for 2 hours in wells coated with recombinant human soluble Fas, 5 μg/ml. The sFasL bound to Fas was detected using a polyclonal anti-human FasL antibody. The mut sFasL binding curve was shifted to the left. n = 3. (C) Jurkat cells were incubated in 96-well plates at a concentration of 1 x 10^4^ cells/well. Different molar ratios of the Fas-activating antibody CH-11 and mut-sFasL were added, and caspase 3/7 activity measured after 5 hours. The mut-sFasL did not inhibit the activity of CH11. Data represent the results from three separate experiments, each done in duplicate, and were analyzed by 2-way ANOVA with Sidak’s post hoc analysis; (A), WT-sFasL compared to mut-sFasL at the same concentrations; (B), comparisons made to no competitor condition for each molecule; (C) Comparisons made to unmixed antibody (Ab). Each dot represents individual data, lines represent means ± SD. * = P < 0.05; ** = P<0.01, ***P<0.001.

We next investigated whether the mut-sFasL attenuated WT-sFasL activity by competing for the Fas receptor. We coated wells with recombinant human Fas at 250 ng/mL and measured binding of a fixed concentration of biotinylated WT-sFasL (70 ng/mL) in the presence of serial 3-fold dilutions of either WT-sFasL or mut-sFasL. As the concentration of non-biotinylated WT-sFasL increased, binding of biotinylated WT-sFasL to Fas decreased, indicating competition for the Fas receptor (Fig 4A). However, increasing concentrations of mut-sFasL had little effect on binding of biotinylated WT-sFasL, except at very high concentrations. This finding was surprising and suggested that the mut-sFasL attenuates WT-sFasL activity by a mechanism other than competition for the receptor. Therefore, we next investigated whether the two different forms of sFasL differ in the ability to bind directly to immobilized Fas. We incubated serial dilutions of either WT-sFasL or mut-sFasL in wells coated with recombinant human Fas, 5 μg/mL, and after washing detected WT-sFasL or mut-sFasL bound to Fas using a polyclonal anti-FasL antibody. The binding curve of mut-sFasL was shifted to the right, indicating decreased binding affinity for Fas. It is possible that the decreased binding affinity could contribute to the lack of competition between the mut-sFasL and WT-sFasL.

To further examine the activity of mut-sFasL at the Fas receptor, we asked whether mut-sFasL would inhibit an antibody capable of binding and activating Fas in viable cells. We measured caspase 3/7 activity in Jurkat cells following 5-hour incubation with the Fas-activating antibody CH11, a control antibody, or different molar ratios of mut-sFasL and antibody. The mut-sFasL did not attenuate the caspase 3/7 activation induced by CH11, and in fact, at very high molar ratios it increased CH11 activity (Fig 4C). Together, these observations suggest that mut-sFasL has low binding affinity for Fas, and that the inhibitory mechanism is likely unrelated to direct antagonism at the receptor level.

### The mut-sFasL forms aggregates with WT sFasL

**Fig 5:**
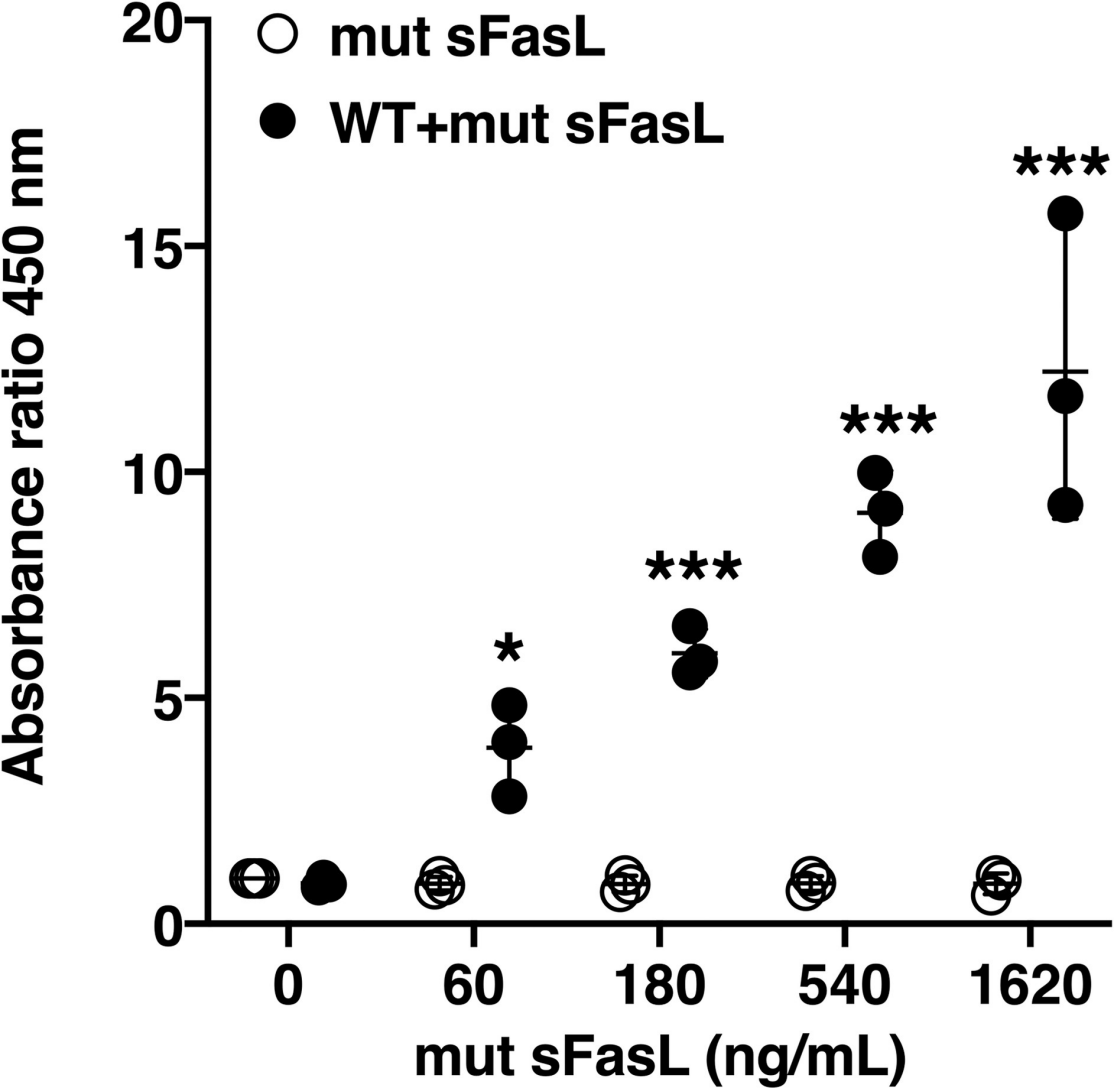
The mut-sFasL forms aggregates with WT sFasL protein. Serial 3-fold dilutions of FLAG tagged mut-sFasL were mixed with biotinylated WT sFasL (60 ng). The mixtures were incubated at room temperature for 15 minutes and applied to streptavidin-coated wells. FLAG tags were detected with HRP anti-FLAG mAb. There was a dose-dependent increase in FLAG signal in wells incubated with the biotinylated WT sFasL and the mut-sFasL-FLAG mixture, indicating formation of complexes of WT sFasL and mut-sFasL. The mut-sFasL-FLAG without biotin did not bind to the streptavidin-coated plates. Data shown as ratio of the absorbance at 450 nm of each experimental condition to that of media only. Data were generated from three separate experiments, each done in duplicate, and were analyzed by 2-way ANOVA with Dunnet’s post hoc analysis; comparisons were made to 0 ng/mL for each condition. Each dot represents individual data, lines represent means ± SD. * = P < 0.05; ** = P<0.01, ***P<0.001.

Previous studies have established that in order to be active, sFasL must form trimers or higher order multimers [14]. Therefore, we tested whether the mut-sFasL forms complexes with WT-sFasL. FLAG-tagged mut-sFasL was mixed with biotinylated WT-sFasL at the same molar ratios used in the experiments shown in Fig 2. The mixture was incubated in streptavidin-coated wells to capture the biotinylated WT-sFasL. After washing unbound material, FLAG was detected using an anti-FLAG antibody, which would detect FLAG-tagged mut-sFasL complexed with biotinylated WT-sFasL. As shown in Fig 5, the FLAG-tagged mut-sFasL by itself did not bind to the plate, even at high concentrations. However, there was a dose-dependent increase in FLAG signal in wells treated with fixed concentrations of biotinylated WT sFasL and increasing concentrations of FLAG-tagged mut sFasL, indicating the formation of WT sFasL:mut-FasL complexes.

### The mut-sFasL attenuates the lung inflammatory response to intratracheal LPS

**Fig 6:**
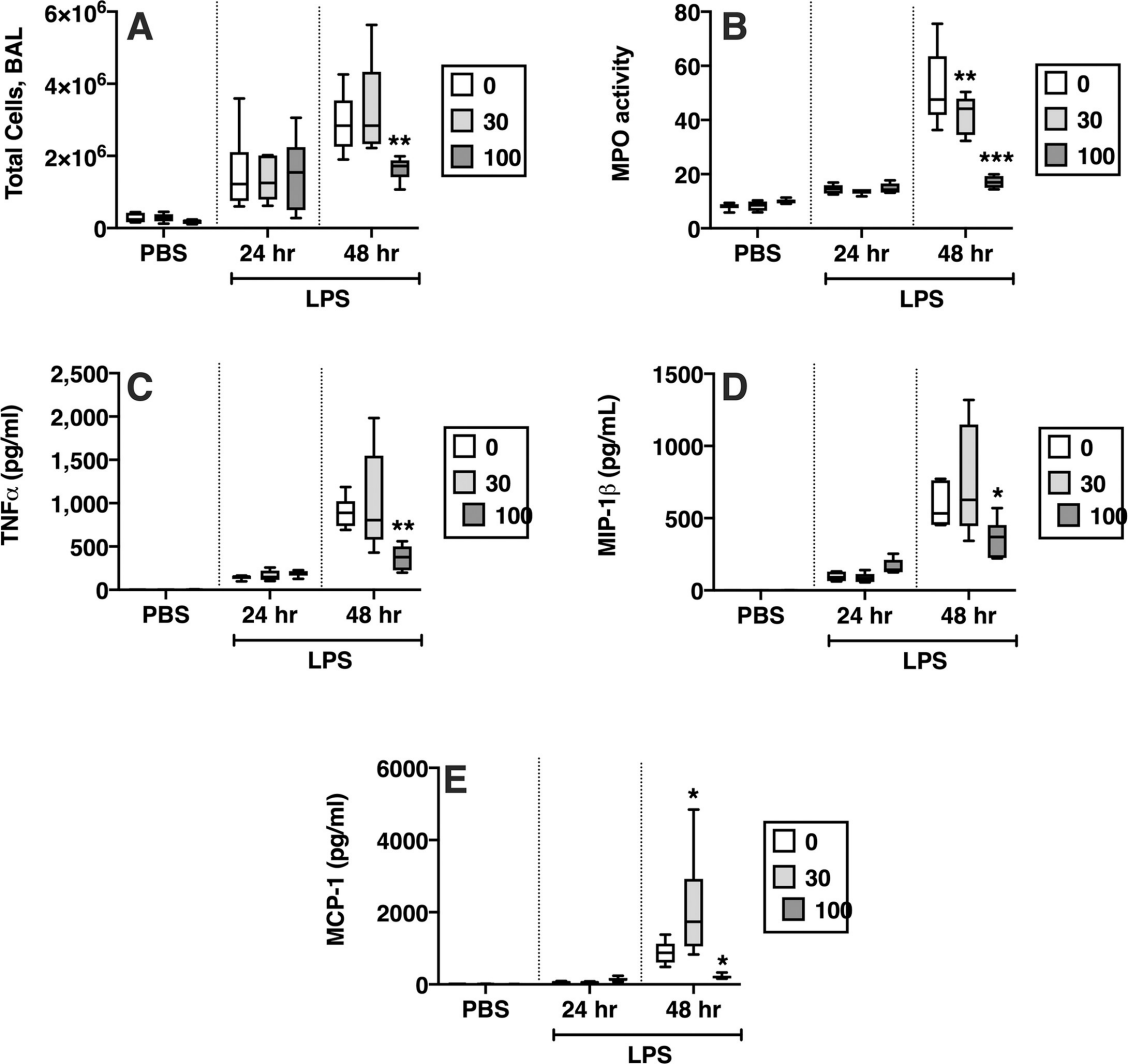
Effects of mut-sFasL on markers of lung inflammation in LPS-treated mice. C57BL/6 mice received oropharyngeal instillations of either PBS or LPS, 2.5 μg/mL. Four hours later some of the mice received oropharyngeal instillations of PBS or mut-sFasL at concentrations of 10 and 100 ng/g and were euthanized 24 hours after the initial LPS/PBS instillations (“early” group). Other mice received the PBS or mut-sFasL (same dose range) 24 hours after LPS/PBS and were euthanized 48 hours after the original LPS instillation (“late” group). N = 6 for all groups. In the “late” group, treatment with 100 ng/g mut-sFasL reduced BAL total cells (A), lung homogenate MPO activity (B), and the BAL concentrations of TNF-α, MIP-1β and MCP-1 (C-E). Data were analyzed by 2-way ANOVA with Dunnet’s post hoc analysis; comparisons made to 0 ng/mL for each condition. Data are reported as box plots, in which the box depicts the 25^th^ to 75^th^ percentiles and the lines the min to max range. * = P < 0.05; ** = P<0.01, ***P<0.001.

Next, we designed an experiment to test whether the mut-sFasL would affect the response of mice to bacterial lipopolysaccharide (LPS), an established model or sterile lung injury. Mice were treated with intratracheal instillations of LPS, 2.5 μg/g, or PBS (“Time = 0”). An “early group” of mice received mut-sFasL after 4 hours and were euthanized at 24 hours. A “late group” received mut-s-FasL at 24 hours followed by euthanasia at 48 hours. We found that in the late group, mut-sFasL led to mild changes in some measures of the inflammatory response (Fig 6). Specifically, in the late group the BAL total cell counts were decreased in mice receiving 100 ng/mL mut-sFasL (Fig 6A). Total lung MPO activity, a measure of total lung neutrophils, also was significantly decreased in the mut-sFasL-treated mice at doses of 30 and 100 ng/mL (Fig 6B). The attenuation of the neutrophil response was associated with a decrease in the BAL concentrations of TNF-α, MIP-1 β and MCP-1 (Fig 6C–6E). The neutrophil chemoattractants KC and MIP-2 were not affected, and there was an increase in IL-1β and MIP-1α (Table 1). Representative images of immunohistochemical staining for LyG (neutrophil marker) and caspase 3 are shown in S5 Fig.

**Table 1:** BAL cytokine concentration.

	PBS			24 hr			48 hr		
	0	30	100	0	30	100	0	30	100
**IL-1β**	0.2 ± 0.0	0.2 ± 0.0	0.2 ± 0.1	11.3 ± 3.4	14.3 ± 10.6	18.6 ± 5.5	24.0 ± 7.4	37.8 ± 23.3	44.5 ± 23.1
**MIP-1α**	0.1 ± 0.05	0.08 ± 0.03	0.29 ± 0.28	54.3 ± 14.4	47.9 ± 10.6	85.3 ± 20.0	109.3 ± 19.4	154.5 ± 78.7	153.9 ± 42.4
**KC**	1.5 ± 1.1	1.2 ± 0.6	1.9 ± 0.9	152.6 ± 32.7	118.4 ± 43.7	154.4 ± 94.3	110.4 ± 44.5	113.2 ± 55.8	136.9 ± 51.9
**MIP-2**	0.1 ± 0.0	0.1 ± 0.0	2.3 ± 2.8	107.7 ± 23.2	78.0 ± 28.1	124.1 ± 29.1	107.5 ± 14.8	146.7 ± 72.7	135.7 ± 29.9

Data shown as means ± standard deviations.

**Fig 7:**
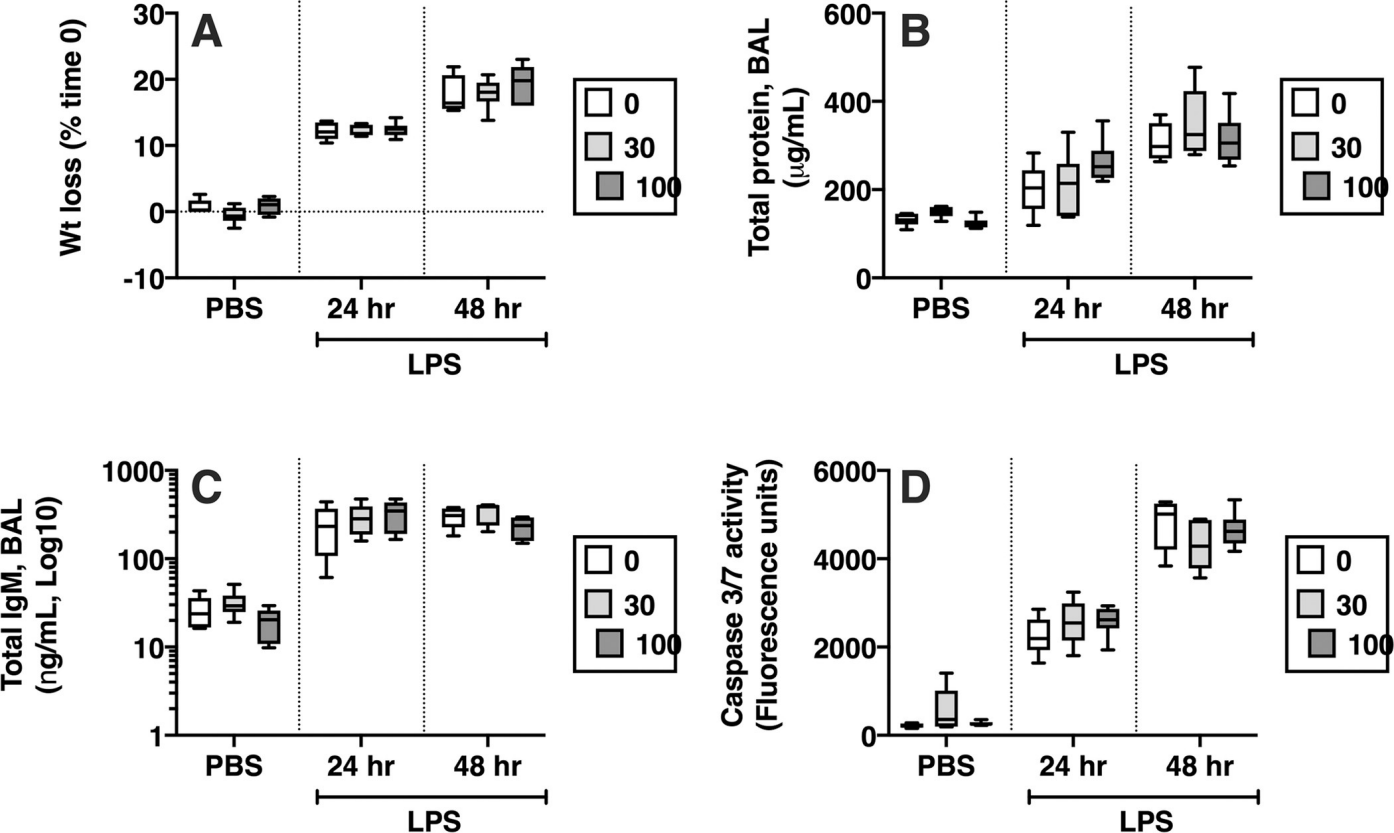
Effects of mut-sFasL in LPS treated mice. C57BL/6 mice received oropharyngeal instillations of either PBS or LPS, 2.5 μg/mL. Four hours later some of the mice received oropharyngeal instillations of PBS or mut-sFasL at concentrations of 10 and 100 ng/g and were euthanized 24 hours after the initial LPS/PBS instillations (“early” group). Other mice received the PBS or mut-sFasL (same dose range) 24 hours after LPS/PBS and were euthanized 48 hours after the original LPS instillation (“late” group). N = 6 for all groups. Weight loss was similar regardless of treatment (A, percent change from initial weight). The administration of mut-sFasL had no effect total BAL protein (B), BAL IgM (C) or total lung homogenate caspase 3/7 activity (D). Data are reported as box plots, in which the box depicts the 25^th^ to 75^th^ percentiles and the lines the min to max range. Data were analyzed by 2-way ANOVA with Dunnett’s post hoc analysis; comparisons were made to 0 ng/mL for each condition.

As expected, the LPS-treated animals lost weight, but the extent of weight loss was not affected by the mut-sFasL (Fig 7A). The mut-sFasL did not lead to an increase in lung apoptosis or permeability measurements in the PBS-treated mice, and it did not affect the LPS-induced increases in lung homogenate caspase-3/7 activity (used as marker for apoptotic activity) or BAL concentrations of total protein and the high molecular weight protein IgM (used as measurements of alveolar barrier permeability) (Fig 7B–7D).

## Discussion

We have previously demonstrated in separate gain-of-function and loss-of-function studies that Fas activation is associated with lung injury in mice, and our findings have been corroborated by other groups [9, 18, 24–26]. We have also shown that the key target cells of sFasL in lung injury are alveolar epithelial cells, which respond to Fas ligation with both apoptosis and cytokine release [9, 12, 15]. The relevance of these findings was demonstrated in studies showing that in humans, sFasL can exist in a long-form, containing a 26-amino acid “stalk” at the N-terminus, and a short form, in which the stalk has been cleaved by metalloproteinases [20]. Importantly only the long form is active, and during ARDS, oxidation of key residues of the stalk region prevent its cleavage, thereby stabilizing the active “long” form [20]. In the present study, we show that mutation of the charged residues of the sFasL stalk region into non-charged alanines results in a mutant protein with attenuated activity that acts as an inhibitor of the naturally occurring protein *in vitro*, and retains mild inhibitory properties *in vivo*.

The Fas/FasL system is composed of Fas (CD95) and its cognate ligand, FasL (CD178). In the lungs, Fas is expressed ubiquitously [11, 12], whereas FasL is expressed in lung epithelial cells [27], neutrophils, macrophages, and lymphocytes [28]. Fas is a type I transmembrane receptor member of the TNF superfamily of proteins [29]. The intra-cytoplasmic domain of Fas can exist in two forms, a “closed” form and an “open” form [30]. The open form of Fas binds the adapter protein FADD via death domain (DD) homotypic interactions, however, the open form is unstable in the absence of FasL. Binding of Fas to multimers of FasL stabilizes Fas in the open form by inducing clustering of Fas in tetramers or larger oligomers [30]. Once FADD binds to the intracellular portion of Fas, a signaling cascade is initiated, leading to activation of the cysteine protease caspase 8, activation of caspase 3, and execution of apoptosis. Thus, three important events in the initiation of Fas signaling are, first, multimerization of FasL, second, binding of FasL to Fas; third, clustering of the Fas/FasL complex and stabilization of the open form of Fas.

Fas ligand is a 281-amino acid, 40 kDa type II transmembrane protein. The N- terminal region is cytoplasmic and the C-terminal domain is extracellular. The extracellular domain is divided in two additional domains: a 144-amino acid TNF-homology domain (THD), which mediates receptor binding and is rich in aromatic and hydrophobic residues, and a 34 amino acid stalk region that links the THD with the transmembrane domain [31]. The extracellular domain can be shed following cleavage by a number of proteases including ADAM10, MMP3 and MMP7, resulting in sFasL [21, 27, 32, 33]. However, the biological role of sFasL has been controversial. Early studies suggested that sFasL lacked bioactivity and in fact could even function as an inhibitor of membrane-bound FasL [34–36]. O’Reilly et al suggested that sFasL retains pro-inflammatory activity, but not pro-apoptotic activity [37]. In contrast, Shudo et al suggested the opposite [38]. Some studies suggested that the stalk region of sFasL was key to its bioactivity [39], and it is certainly involved in sFasL oligomerization [31]. As mentioned earlier, we found that the bioactivity of sFasL depends of the length of its stalk region, which in vivo exists in two forms: an inactive short form, containing amino acids 129–281, and a bioactive long form, containing amino acids 103–281 [20]. The long form predominates in the BAL of patients with ARDS, is stabilized by oxidation, and accounts for most of its pro-apoptotic activity.

We decided to investigate the contribution of the stalk region of sFasL to sFasL bioactivity. Interestingly, sFasL binds Fas at the C-terminal side of the protein, suggesting that the mechanism behind the difference in activity between the long and short forms could be a conformational change in the tertiary structure interfering with one of the three steps of Fas signaling described above (multimerization, binding or receptor clustering). Thus, we changed the 8 charged residues of the stalk region to the non-charged amino acid alanine in order to reduce ionic binding of the stalk region to putative binding partners. The resultant mutant protein (mut-sFasL) had significantly attenuated activity compared to WT sFasL and acted as an inhibitor of the WT protein. Interestingly, the prediction model of this mutation suggests that changing the charged residues to alanine results in conformational changes in the stalk region of the trimeric form of the sFasL (S1B Fig). This computer modeling of sFasL and of the mutant sFasL has excellent concordance with the predicted structure of “short” sFasL described in the original work characterizing Fas-FasL interactions [40]. In this work, the authors noted that the “short” sFasL trimers did not bind Fas in a stable manner in the absence of glycosylation. Based on these observations and our computer model in which the spatial arrangement of the stalk region is altered, we speculate the stalk region may play a role in glycosylation and Fas interaction. We emphasize this is our prediction based on computer modeling and prior published work. Future studies confirming the crystal structure of the proteins and their roles in Fas-FasL interaction will be needed.

Our experiments showed that the inhibition was unlikely a result of direct competition for the receptor or increased binding affinity. The observation that the mut-sFasL did not affect the activity of a Fas-binding antibody further suggested that the inhibitory mechanism was unlikely at the receptor level, and may involve mut-sFasL interaction with the WT sFasL protein prior to receptor engagement. Capture of the mut-sFasL by WT sFasL protein provided additional supportive evidence that mut-sFasL forms heteromultimeric complexes with WT sFasL. We speculate the inhibitory action of mut-sFasL is not by direct antagonism of the receptor. Rather, sFasL heterodimerization with the mutant protein is likely responsible for the loss of activity of WT sFasL. Confirmation of the inhibitory mechanism will require further studies to elucidate the precise structure of these complexes and their interactions with Fas.

Some experiments in this report were conducted using Jurkat cells as a known responder of Fas-FasL pathway. However, our primary biological focus is on the effect of sFasL on airway epithelium. We previously published work on the functional effect of activating the Fas pathway in the murine epithelial cell line La4 [41]. In that study, we demonstrated the activation of the Fas signaling pathway by the activating antibody Jo2 increased cell death and LA4 monolayer permeability in a dose dependent manner. Furthermore, inhibition of caspase activity by the pan-caspase inhibitor zVAD reversed cell death and monolayer permeability induced by Jo2, implicating caspase activity in the functional consequences of Fas activation. These findings have been further confirmed and elaborated in human pulmonary alveolar epithelial cells (HPAEpiC) in our recent work [42]. HPAEpiC exposed to the long form of sFasL induced caspase 3, with subsequent increases in permeability of HPAEpiC monolayers followed by cell death. We further showed that tight junction proteins ZO-1 and occludin were down-regulated with Fas activation. Importantly, pretreatment of HPAEpiCs with caspase 3-specific inhibitor zDEVD.fmk reversed the effect of sFasL on HPAEpiC monolayer permeability and ZO-1/occludin expression, demonstrating the functional effect is mediated through caspase 3. Now, we present evidence demonstrating the inhibitory effect of mut sFasL on caspase 3/7 induction in epithelial cells. WT:mut sFasL molar ratios of 1:27 and 1:81 significantly reduced caspase 3/7 activity as well as Annexin V translocation on SAECs in this study, consistent with findings in our previous work.

Given that the mut-sFasL attenuated WT-sFasL activity *in vitro*, we tested whether it could attenuate lung injury *in vivo*. There are several lines of evidence suggesting that targeting the Fas/FasL system could be of particular importance as a potential therapeutic for acute lung injury, because it affects multiple key pathways leading to two of the key features of lung injury: disruption of the alveolar/epithelial barrier and inflammation. Disruption of the alveolar epithelial barrier occurs by at least two mechanisms: First, although Fas is ubiquitously expressed in the lungs, adoptive transfer studies and macrophage depletion studies have confirmed that Fas activation specifically targets non-myeloid cells, leading to caspase 3/7 activation, apoptosis and loss of membrane integrity [8, 15, 16]. In addition to this structural injury, Fas activation also attenuates the expression of alveolar epithelial tight junction proteins, a caspase-dependent effect that occurs even in the absence of apoptosis [42]. Combined, both mechanisms lead to impairment of the epithelial barrier, as well as reversible disruption of alveolar fluid clearance [41]. In addition to its effects on alveolar epithelial barrier, Fas activation is also a powerful inducer of pro-inflammatory cytokine release, by a mechanism involving activation of the MAP-kinase pathway and transcription factors such as AP-1 [13, 43]. *In vitro*, this effect is seen in myeloid cells and in alveolar epithelial cells, but *in vivo*, only the presence of Fas in the alveolar epithelium is required for induction of lung inflammation by sFasL [13]. Importantly, studies using sterile and non-sterile models of injury and Fas-deficient animals suggest that the contribution of the Fas/FasL system to lung inflammation is not trivial [18, 24, 25]. Thus, inhibition of the Fas/FasL system could play an important role as a novel therapeutic strategy for lung injury.

We chose a model of lung injury induced by bacterial lipopolysaccharide as a stereotypical, well-defined one-hit model that would allow evaluation of one-time administration of the mut-sFasL. The mutant protein was administered either 4 hours after LPS, to test its effects during the very early phases of lung injury, or 24 hours after LPS, to evaluate its effects at a point when lung injury is fully established. While early administration had no measurable effect, in the late group mut-sFasL reduced some parameters of inflammation. The dominant effect was a reduction in total BAL cells and whole lung MPO (a measurement of the total lung content of neutrophils), as well as the cytokines TNF-α, MCP-1 and MIP-1β (CCL4). Taken together, the 24 hr data show a reduction in lung inflammation, with reduced TNFa and MCP-1 and a reduction in lung neutrophil content. Surprisingly, the main murine neutrophil chemoattractants KC (CXCL1) and MIP-2 (CXCL2) were not affected. This pattern of attenuated neutrophilic response with unchanged concentrations of KC and MIP-2 and no change in permeability markers is very similar to that seen in Fas-deficient *lpr* mice exposed to LPS and mechanical ventilation [18]. In that study, we found that the lack of functional Fas was associated with decreased deposition of anti-KC:KC immune complexes in the lungs, which are known to enhance the inflammatory response [44, 45]. Further studies will investigate the mechanism whereby the mut-sFasL attenuates the neutrophil response and affects cytokine release; however, the available data support the hypothesis that inhibition of the Fas/FasL system by a sFasL with mutated stalk region is not injurious in normal lungs and can have a biological effect in injured lungs *in vivo*, reproducing the phenotype of Fas-deficient *lpr* mice in sterile lung injury.

In summary, we have demonstrated that mutation of the charged amino acids of the stalk region of sFasL to non-charged amino acids markedly attenuates the biological activity of sFasL. The mutated protein likely heterodimerizes with wild type sFasL and inhibits its activity *in vitro*. The mutated protein did not have important pro-injury or pro-inflammatory effects at any dose when administered 24 hours after intratracheal LPS, confirming the critical role of the stalk region in the bioactivity of sFasL *in vivo*. In addition, the mut-sFasL was a weak inhibitor of the inflammatory response. Further studies are required to establish the optimal design of a sFasL inhibitor *in vivo*, but the present work supports a focus on alterations of the stalk region or development of inhibitors that bind to the stalk region. We conclude that changes in the structure of the stalk region of sFasL interfere with its function and provide promising targets in the design of novel therapeutic agents directed at the Fas/FasL system.

## Supporting information

S1 Fig Fas activation pathways in the lungs. In alveolar epithelial cells, Fas activation can lead to apoptosis, but also to cytokine release via adapter proteins such as MyD88.
(PDF)

S2 Fig DNA sequencing data for the FasL constructs. DNA sequence of wild type sFasL construct (top row) and eight different clones (two rows per clone, each row showing a different forward primer). The mutant sFasL clone used in the study is Clone 1, and its sequence is presented on the 2nd and 3rd rows (Eurofin Custom Sequencing Service).
(PDF)

S3 Fig Predicted protein structures. The top row shows monomeric forms (top) and the bottom row trimeric forms. On the left column, the long (wild type) sFasL monomer shows the stalk region as an alpha helix (blue) which is absent in the short sFasL, lacking the stalk region (right column). Spatial arrangement of the stalk region in the trimeric form of the long sFasL (bottom panel, left) is highlighted by the white elipses. Mutation of all the charged amino acids in the stalk region is predicted to rotate the spatial arrangement of the stalk region (white elipses) in the trimeric form of mutant sFasL (bottom panel, middle) compared to long sFasL (bottom panel, left). Images of predicted protein structures were generated using Rosetta Molecular Modeling Suite (https://robetta.bakerlab.org).
(PDF)

S4 Fig Effects of mut-sFasL on caspase 3/7 activity. Effect of increasing concentrations of mut-sFasL on caspase 3/7 activity (white violin bars) or in combination with a fixed concentration of WT sFasL. We observed that only at the highest dose of 2430 ng/mL, corresponding to the 1:81 WT:mut sFasL molar ratio in Fig 3, the mut-sFasL leads to intrinsic caspase-3/7 activation.
(PDF)

S5 Fig Representative immunohistochemistry. Images of lung sections from mice treated with LPS+PBS (top panels) and LPS+mut sFasL (bottom panels) at 48 hr post-LPS instillation. Staining for the neutrophil marker Ly6G (left) and for Caspase-3 (right) are shown. (Bar = 100 μm).
(PDF)

S1 Text Immunohistochemistry protocol. (PDF)

S1 Data Raw data Kajikawa et al. This is a spreadsheet file containing the source data for the figures shown in the manuscript.
(XLSX)
