# Supplementary material for: The bioactivity of soluble Fas ligand is modulated by key amino acids of its stalk region
Source: PLoS One. 2021 Jun 17;16(6):e0253260. doi: 10.1371/journal.pone.0253260 (PMC8211282; doi:10.1371/journal.pone.0253260)
Supplement: S1 Fig — In alveolar epithelial cells, Fas activation can lead to apoptosis, but also to cytokine release via adapter proteins such as MyD88. (PDF) [file pone.0253260.s001.pdf]

S1 Fig

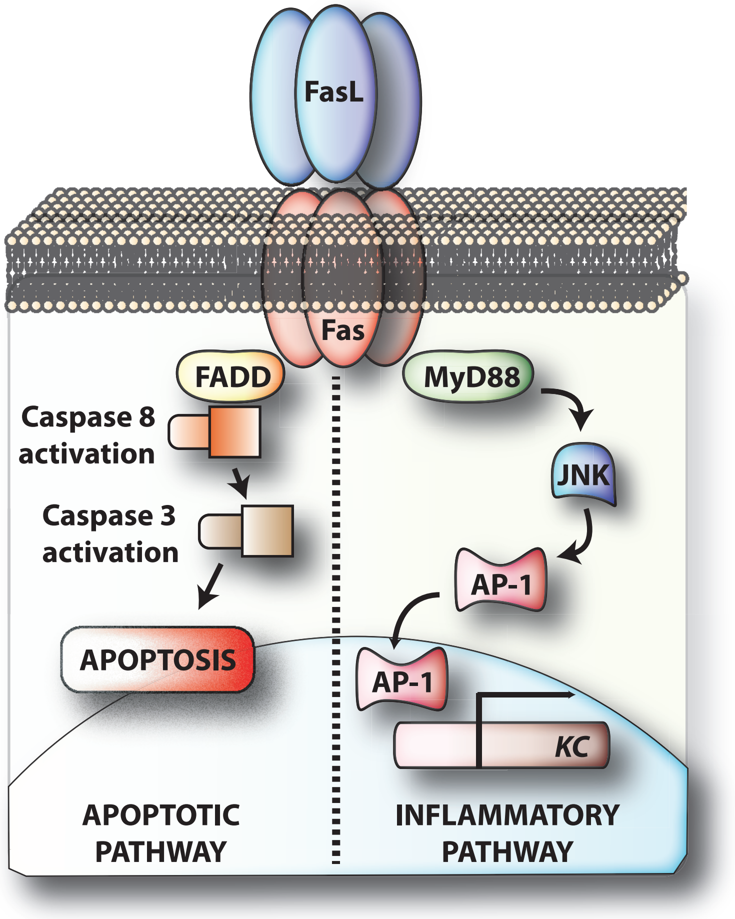

S1 Fig: Fas activation pathways in the lungs. In alveolar epithelial cells, Fas activation can lead to apoptosis, but also to cytokine release via adapter proteins such as MyD88.
