# Supplementary material for: The bioactivity of soluble Fas ligand is modulated by key amino acids of its stalk region
Source: PLoS One. 2021 Jun 17;16(6):e0253260. doi: 10.1371/journal.pone.0253260 (PMC8211282; doi:10.1371/journal.pone.0253260)
Supplement: S2 Fig — DNA sequence of wild type sFasL construct (top row) and eight different clones (two rows per clone, each row showing a different forward primer). The mutant sFasL clone used in the study is Clone 1, and its sequence is presented on the 2nd and 3rd rows (Eurofin Custom Sequencing Service). (PDF) [file pone.0253260.s002.pdf]

S2 Fig

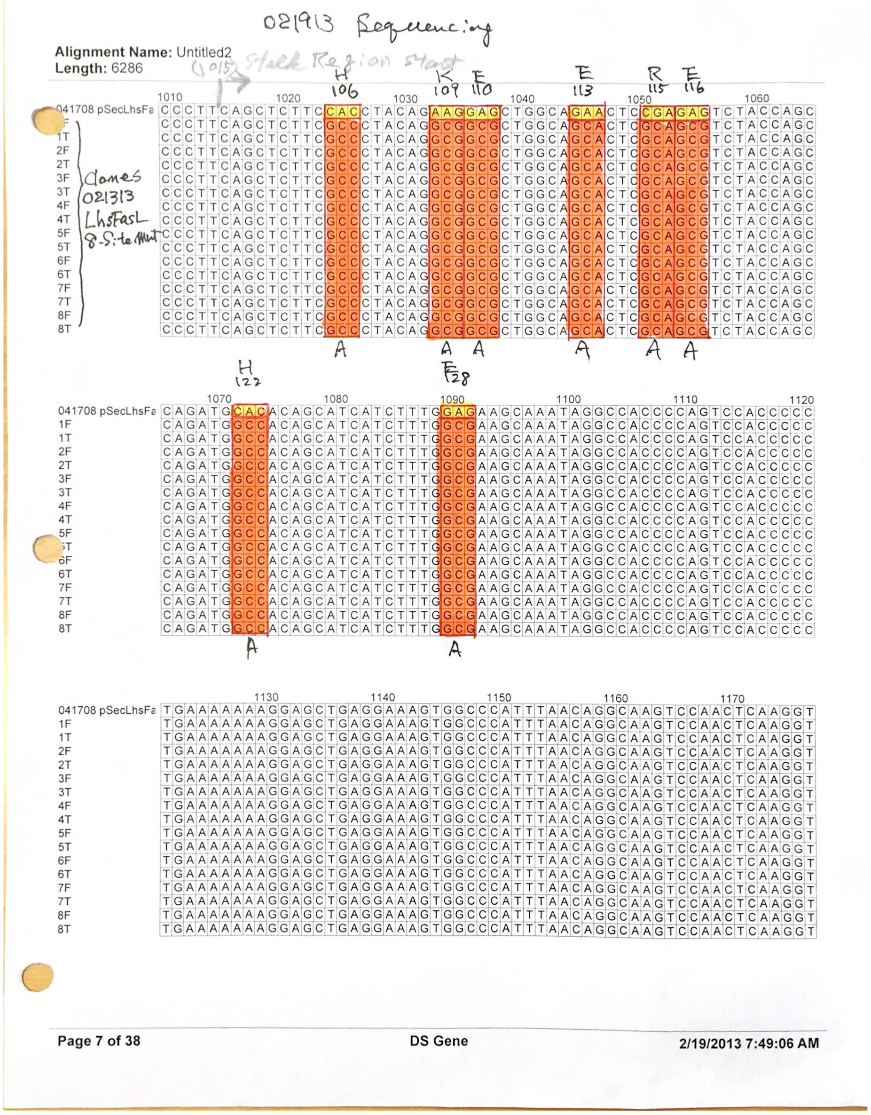

S2 Fig: **DNA sequencing data for the FasL constructs**. DNA sequence of wild type sFasL construct (top row) and eight different clones (two rows per clone, each row showing a different forward primer). The mutant sFasL clone used in the study is Clone 1, and its sequence is presented on the 2nd and 3rd rows (Eurofin Custom Sequencing Service) .
