# Supplementary material for: The bioactivity of soluble Fas ligand is modulated by key amino acids of its stalk region
Source: PLoS One. 2021 Jun 17;16(6):e0253260. doi: 10.1371/journal.pone.0253260 (PMC8211282; doi:10.1371/journal.pone.0253260)
Supplement: S3 Fig — The top row shows monomeric forms (top) and the bottom row trimeric forms. On the left column, the long (wild type) sFasL monomer shows the stalk region as an alpha helix (blue) which is absent in the short sFasL, lacking the stalk region (right column). Spatial arrangement of the stalk region in the trimeric form of the long sFasL (bottom panel, left) is highlighted by the white elipses. Mutation of all the charged amino acids in the stalk region is predicted to rotate the spatial arrangement of the stalk region (white elipses) in the trimeric form of mutant sFasL (bottom panel, middle) compared to long sFasL (bottom panel, left). Images of predicted protein structures were generated using Rosetta Molecular Modeling Suite (https://robetta.bakerlab.org). (PDF) [file pone.0253260.s003.pdf]

**S3 Fig**

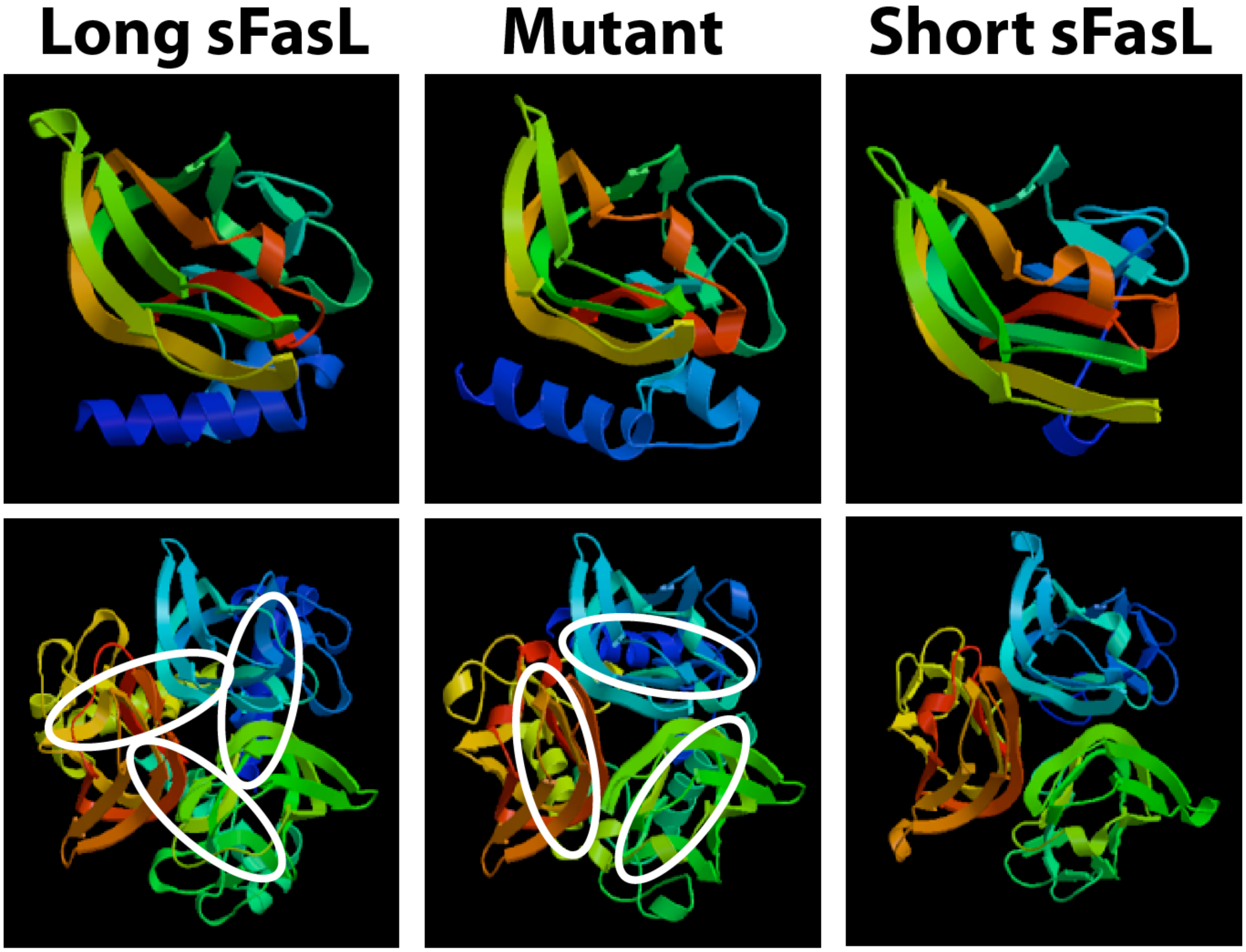


**S3 Fig: Predicted protein structures.** The top row shows monomeric forms (top) and the bottom row trimeric forms. On the left column, the long (wild type) sFasL monomer shows the stalk region as an alpha helix (blue) which is absent in the short sFasL, lacking the stalk region (right column). Spatial arrangement of the stalk region in the trimeric form of the long sFasL (bottom panel, left) is highlighted by the white elipses. Mutation of all the charged amino acids in the stalk region is predicted to rotate the spatial arrangement of the stalk region (white elipses) in the trimeric form of mutant sFasL (bottom panel, middle) compared to long sFasL (bottom panel, left). Images of predicted protein structures were generated using Rosetta Molecular Modeling Suite (https://robetta.bakerlab.org).
