# Supplementary material for: The bioactivity of soluble Fas ligand is modulated by key amino acids of its stalk region
Source: PLoS One. 2021 Jun 17;16(6):e0253260. doi: 10.1371/journal.pone.0253260 (PMC8211282; doi:10.1371/journal.pone.0253260)
Supplement: S4 Fig — Effect of increasing concentrations of mut-sFasL on caspase 3/7 activity (white violin bars) or in combination with a fixed concentration of WT sFasL. We observed that only at the highest dose of 2430 ng/mL, corresponding to the 1:81 WT:mut sFasL molar ratio in Fig 3, the mut-sFasL leads to intrinsic caspase-3/7 activation. (PDF) [file pone.0253260.s004.pdf]

S4 Fig

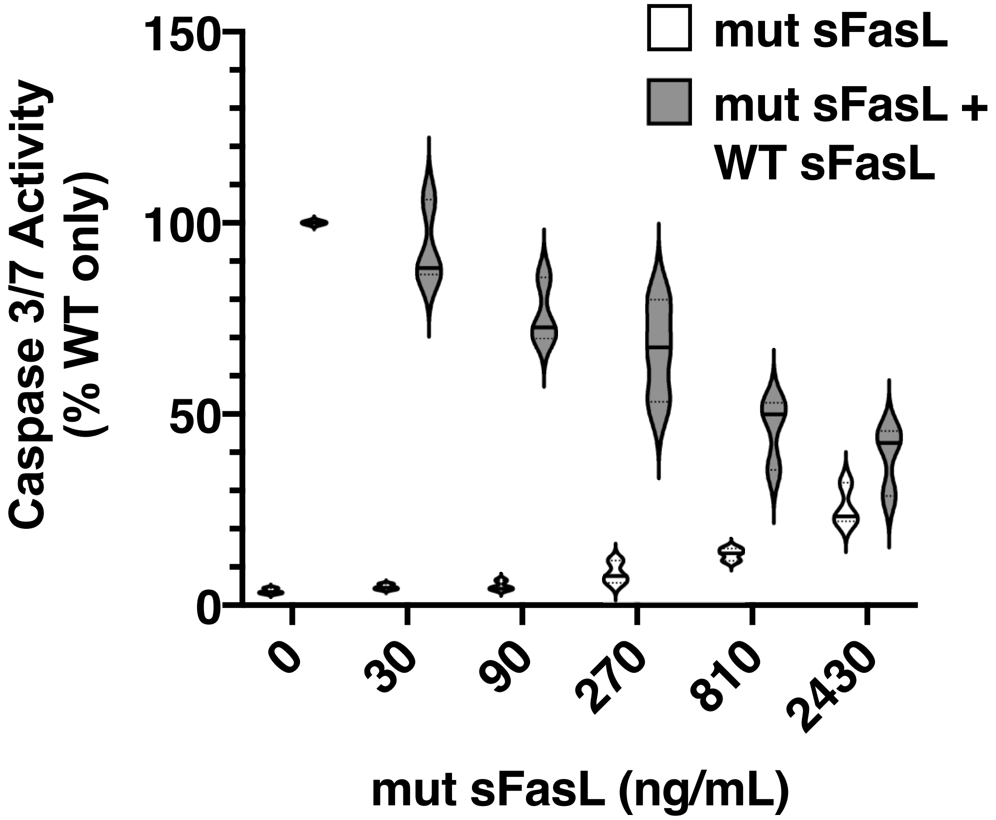

S4 Fig: Effects of mut-sFasL on caspase 3/7 activity: Effect of increasing concentrations of mut-sFasL on caspase 3/7 activity (white violin bars) or in combination with a fixed concentration of WT sFasL. We observed that only at the highest dose of 2430 ng/mL, corresponding to the 1:81 WT:mut sFasL molar ratio in Fig 3, the mut-sFasL leads to intrinsic caspase-3/7 activation.
