# Supplementary material for: The bioactivity of soluble Fas ligand is modulated by key amino acids of its stalk region
Source: PLoS One. 2021 Jun 17;16(6):e0253260. doi: 10.1371/journal.pone.0253260 (PMC8211282; doi:10.1371/journal.pone.0253260)
Supplement: S1 Text — (PDF) [file pone.0253260.s006.pdf]

S1 Text

Immunohistochemistry protocols

Immunohistochemistry for Ly6G and cleaved caspase-3 was performed using standardized protocols developed at the University of Washington's Histology and Imaging Core (http://www.uwhistologyandimaging.org). For Ly6G immunohistochemistry, antigen retrieval was performed using EDTA for 10 minutes. Slides were than exposed to the primary rat monoclonal anti Ly-6G, Clone 1A8. (BioLegend, San Diego, CA Cat. No. 127602) at a 1:2,000 dilution. The caspase 3 immunohistochemistry was performed antigen retrieval with EDTA for 10 minutes before incubating with the primary rabbit polyclonal anti-cleaved caspase 3 (BioCare Medical, Pacheco, CA Cat. No. CP229B). All primary antibodies were diluted in Leica Primary antibody diluent for 30 minutes at room temperature. Blocking prior to addition of the primary antibody consisted of 10% Normal Goat Serum in TBS buffer for 20 minutes at room temperature. The Ly6G primary antibody was followed by a rabbit anti-rat IgG(H+L), mouse adsorbed, unconjugated secondary (Vector, Burlingame, CA, #AI-4001, lot #ZF-0513) at 1:300 in 5% NGS and TBS for 8 minutes at room temperature. The cleaved caspase 3 antibodies were incubated with goat anti-rabbit poly-HRP polymerized secondary detection (Leica Cat No DS9800) for 8 minutes at room temperature. Negative controls were performed using purified Rabbit IgG (R&D Systems, Minneapolis, MN, #AB-105-C; lot #ER1314071) at 1:1000 or purified Rat IgG2b Isotype (BD Pharmingen, San Jose, CA, #553986) at 1:100 in Leica primary antibody diluent for 30 minutes at room temperature. Additional blocking for endogenous peroxidase was then performed using Leica peroxide block (3% H2O2/Leica Cat No DS9800) for 5 minutes at room temperature. Sections were then incubated with Leica Bond Mixed Refine DAB substrate detection for 10 minutes at room temperature. (Leica Cat No DS9800). Antibody complexes were visualized using Leica Bond Mixed Refine (DAB, 3,3'-diaminobenzidine) detection 2X for 10 minutes at RT (Leica Cat No DS9800). Tissues were counterstained with hematoxylin counterstain for 4 minutes followed by two rinses in water. Slides were then dehydrated through 100% ETOH, cleared in Xylene and mounted with synthetic resin mounting medium and a #1.5 coverslip.
